# Construction of a novel necroptosis-related lncRNA signature for prognosis prediction in esophageal cancer

**DOI:** 10.1186/s12876-022-02421-8

**Published:** 2022-07-15

**Authors:** Yang Liu, Hongyu Hao, Lin Kang, Guona Zheng, Xiaowan Guo, Bingjie Li, Huanfen Zhao, Han Hao

**Affiliations:** 1 Department of Pathology, Hebei General Hospital, Shijiazhuang, China; 2 Department of Neurology, Hebei General Hospital, Shijiazhuang, China; 3 Department of Radiology, Hebei General Hospital, Shijiazhuang, China; 4 Department of Pharmacology, The Key Laboratory of New Drug Pharmacology and Toxicology, Center of Innovative Drug Research and Evaluation, Hebei Medical University, Shijiazhuang, China

**Keywords:** EC, Necroptosis, lncRNA, Prognosis, Immune

## Abstract

**Background:**

Esophageal cancer (EC), one highly malignant gastrointestinal cancer, is the 6th leading cause of cancer-related deaths worldwide. Necroptosis and long non-coding RNA (lncRNA) play important roles in the occurrence and development of EC, but the research on the role of necroptosis-related lncRNA in EC is not conclusive. This study aims to use bioinformatics to investigate the prognostic value of necroptosis-related lncRNA in EC.

**Methods:**

Transcriptome data containing EC and normal samples, and clinical information were obtained from the Cancer Genome Atlas database. 102 necroptosis-related genes were obtained from Kanehisa Laboratories. Necroptosis-related lncRNAs were screened out via univariate, multivariate Cox and the least absolute shrinkage and selection operator regression analyses to construct the risk predictive model. The reliability of the risk model was evaluated mainly through quantitative real-time PCR (qRT-PCR), the receiver operating characteristic (ROC) curves and the constructed nomogram. KEGG pathways were explored in the high- and low-risk groups of EC patients via gene set enrichment analyses (GSEA) software. Immune microenvironment and potential therapeutic agents in risk groups were also analyzed.

**Results:**

A 6 necroptosis-related lncRNAs risk model composed of AC022211.2, Z94721.1, AC007991.2, SAMD12-AS1, AL035461.2 and AC051619.4 was established to predict the prognosis level of EC patients. qRT-PCR analysis showed upregulated Z94721.1 and AL035461.2 mRNA levels and downregulated AC051619.4 mRNA level in EC tissues compared with normal tissues. According to clinical characteristics, the patients in the high-risk group had a shorter overall survival than the low-risk group. The ROC curve and nomogram confirmed this model as one independent and predominant predictor. GSEA analysis showed metabolic and immune-related pathways enriched in the risk model. Most of the immune cells and immune checkpoints were positively correlated with the risk model, mainly active in the high-risk group. For the prediction of potential therapeutic drugs, 16 compounds in the high-risk group and 2 compounds in the low-risk group exhibited higher sensitivity.

**Conclusions:**

Our results supported the necroptosis-related lncRNA signature could independently predict prognosis of EC patients, and provided theoretical basis for improving the clinical treatment of EC.

**Supplementary Information:**

The online version contains supplementary material available at 10.1186/s12876-022-02421-8.

## Background

Esophageal cancer (EC), one of the common malignancies of the upper gastrointestinal system, is currently listed as the 8th most common cancer in the world [1, 2]. In China, EC ranks fifth in the incidence of malignant tumors with 287,000 new cases and 211,000 deaths every year, especially in North China [3, 4]. The early symptoms of EC are not obvious, and most patients seek medical treatment with dysphagia and other clinical symptoms, at this time the lesions have been in the advanced stage, which is the main factor of poor prognosis and low survival rate of EC [5]. Thus it is urgent to explore molecular markers related to EC and apply them to early diagnosis, prognosis assessment and clinical treatment.

Long non-coding RNA (lncRNA), a class of endogenous RNA with more than 200 nucleotides, regulates the phenotypes of various malignant tumors through epigenetic modification, RNA decay and transcriptional regulation. It is involved in the biological processes of tumor cell proliferation, differentiation, migration, invasion and so on. lncRNAs usually have high tissue or cell specificity and may become novel biomarkers for tumor diagnosis and potential therapeutic targets [6, 7]. Studies have found a variety of lncRNAs exhibit abnormal expressions and play an important role in the occurrence and development of EC, which can promote or inhibit cancer [8].

In multicellular organisms, the dynamic balance between cell proliferation and cell death is a necessary condition to maintain the homeostasis in vivo, and is also an important condition for the growth and development of organisms. The incidence of malignant tumors will be greatly increased when producing excessive cell proliferation and inhibiting cell death in the body [9]. Traditionally, cell death has been divided into regulatory and non-regulatory types. However, in recent years, it has been found that cell necrosis can also be regulated by genetic material like apoptosis which is a new mechanism of cell death, it is also called necroptosis controlled by genes but shows pathological features of necrosis in cell morphology, and its regulation process doesn’t depend on caspase. A large number of studies have proved that necroptosis or programmed necrosis plays an important role in the occurrence, development, invasion, metastasis and drug resistance of malignant tumors [10, 11]. At present, the mechanism of necroptosis in EC regulation remains unclear, and the role of necroptosis-related lncRNA in EC is even more inconclusive. This study aims to use bioinformatics to investigate the effect of necroptosis-related lncRNA on prognosis prediction for EC patients and discover new targets for treating EC.

## Methods

### Data collection

Transcriptome data containing 171 samples (160 EC datasets including 3 types of adenomas and adenocarcinomas, squamous cell neoplasms, and cystic, mucinous and serous neoplasms, and 11 normal datasets) and clinical information containing 185 EC patients were obtained from the Cancer Genome Atlas (TCGA) database (https://portal.gdc.cancer.gov/repository). Data for 102 necroptosis-related genes were obtained from map04217 in Kanehisa Laboratories (www.kegg.jp/kegg/kegg1.html) [12], in which 84 necroptosis-related genes were expressed in EC after screening from transcriptome data via ‘limma’ R package. Next, we explored 412 necroptosis-related lncRNAs after screening by ‘limma’ R package (Log_2_ fold change (FC) > 1, false discovery rate (FDR) < 0.05, and *p* < 0.05) from 654 differentially expressed lncRNAs in the transcriptome data of EC. The interaction network diagram between necroptosis-related genes and lncRNAs was constructed by ‘igraph’ R package. Heat map and volcano plot of differential genes were drawn by ‘pheatmap’ and ‘GGploT2’ R packags.

### Identification of necroptosis-related lncRNAs

We used univariate Cox proportional hazard regression analysis to screen necroptosis-related lncRNA related to overall survival (*p* < 0.05) based on the survival time and survival state of EC patients in the TCGA database. Forest plot and heat map of necroptosis-related lncRNAs were constructed via ‘glmnet’ and ‘pheatmap’ R packages. The least absolute shrinkage and selection operator (Lasso) regression was performed with tenfold cross-validation, *p* = 0.05 as well as run for 1000 cycles by ‘survival’, ‘caret’ and ‘glmnet’ R packages. For each cycle, a random stimulation was set up 1000 times in order to prevent overfitting. Sankey diagram was made by ‘ggplot2’ and ‘ggalluvial’ R packages to visualize the mutually regulated connection between necroptosis-related genes and lncRNAs.

### Construction and verification of the risk signature

The risk prediction model was constructed after screening necroptosis-related lncRNAs via multivariate Cox proportional hazard regression analysis (all *p* < 0.05). The model formula was as follows: risk score = $\sum\nolimits_{i = 1}^{n} {coef(i) \times expr(i)}$, where *n*, coef, and expr represented the number of lncRNAs, the regression coefficient of multivariate Cox regression analysis for each lncRNA and each lncRNA expression level, respectively. We randomized all samples into train (N = 80), test (N = 79) and entire (N = 159) sets by “caret” R package, and acccording to the median risk score, the high- and low-risk groups were established. Overall survival, risk score and survival status between the high- and low-risk groups were analyzed to evaluate the prognostic value of the prediction model by ‘survival’, ‘survminer’ and ‘pheatmap’ R packages. The 1-, 2- and 3-year time-dependent receiver operating characteristics (ROC) curves of the model were plotted via the risk score by ‘survival’, ‘survminer’ and ‘timeROC’ R packages.

### Analysis of the risk signature as an independent predictor of prognosis

Univariate and multivariate Cox regression analyses were used to evaluate whether the risk score and clinical features, including age, gender, tumor grade and tumor stage were independent variable factors. Forest plot was drawn by ‘survival’ R package. The ROC curves were made to compare the different factors in the predictive performance by ‘survival’, ‘survminer’ and ‘timeROC’ R packages.

### Quantitative real-time PCR (qRT-PCR)

Total RNA was extracted from EC patients’ tumors and adjacent tissues obtained from Hebei General Hospital using RNAiso Plus total RNA extraction reagent (Takara, Japan). cDNA was synthesized using a PrimeScript RT reagent Kit with gDNA Eraser (Takara, Japan). The reaction conditions were as follows: 37 °C for 15 min, 85 °C for 5 s and 4 °C for termination. The real-time PCR was performed on the Bio-Rad CFX 96 Real-time PCR system (BIO-RAD, USA) using SYBR Premix Ex Taq Real-Time PCR Kit (Takara, Japan) and specific primers (Z94721.1, Forward: CCAAAACAACACTGCCCGAG, Reverse: GCTGACCGATTGACCAGACA; AL035461.2, Forward: AGGTGACTCGCTGCATCATT, Reverse: CCGTGTGGGACACTTACTCG; AC051619.4, Forward: GACACTTCTGGTTGGGGCAT, Reverse: CAACTCTCATGTGCAGGGCA). The reaction conditions were one cycle of initial denaturation at 95 °C for 3 min, followed by 35 cycles of 95 °C for 30 s, 60 °C for 30 s. The 2−ΔΔCq method was used to calculate gene expression. The differences were tested by Two-Sample *t*-Test.

### Construction of nomogram and calibration

We used the data of risk score, age, gender, tumor grade and tumor stage to construct a nomogram for the 1-, 2- and 3-year overall survival. According to the status of each EC patient, the points corresponding to each factor were added up, and the survival rates of 1-, 2- and 3- year could be predicted by the total points. And correction curves based on the Hosmer–Lemeshow test to illustrate whether the prediction outcome showed good consistence with the practical by ‘survival’, ‘regplot’ and ‘rms’ R packages.

### GSEA analyses

We used the gene set enrichment analyses (GSEA) 4.2.3 software to identify the significantly enriched KEGG pathways between the high- and low-risk groups based on the criterion: NOM *p value* < 0*.*05 and |NES|> 1.5.

### Tumor immunology

To investigate the correlation between the risk signature and tumor immune microenvironment, the infiltration values for TCGA-EC dataset samples were calculated through 7 algorithms: XCELL [13], TIMER [14], QUANTISEQ [15], MCPCOUNTER [16], EPIC [17], CIBERSORT-ABS [18] and CIBERSORT [19]. The ‘limma’, ‘scales’, ‘ggplot2’, ‘tidyverse’, ‘ggpubr’ and ‘ggtext’ R packages were performed in analyzing the correlation between immune cells and the risk model, and the results were shown in a bubble chart. Single sample gene set enrichment analysis (ssGSEA) was used to analyze the related function rating of immune function between the high- and low-risk groups by ‘limma’, ‘ggpubr’ and ‘reshape2’ R packages. Besides, we also evaluated tumor immune scores and immune checkpoints activation between high- and low-risk groups by ‘ggpubr’ and ‘ggplot2’ R packages.

### Exploration of potential therapeutic agents

The ‘pRRophetic’ R package was applied to predict therapeutic compounds determined by the half-maximal inhibitory concentration (IC50) of each EC patient on Genomics of Drug Sensitivity in Cancer (GDSC) (https://www.cancerrxgene.org/).

## Results

### Identification of necroptosis-related lncRNAs in EC

**Fig. 1:**
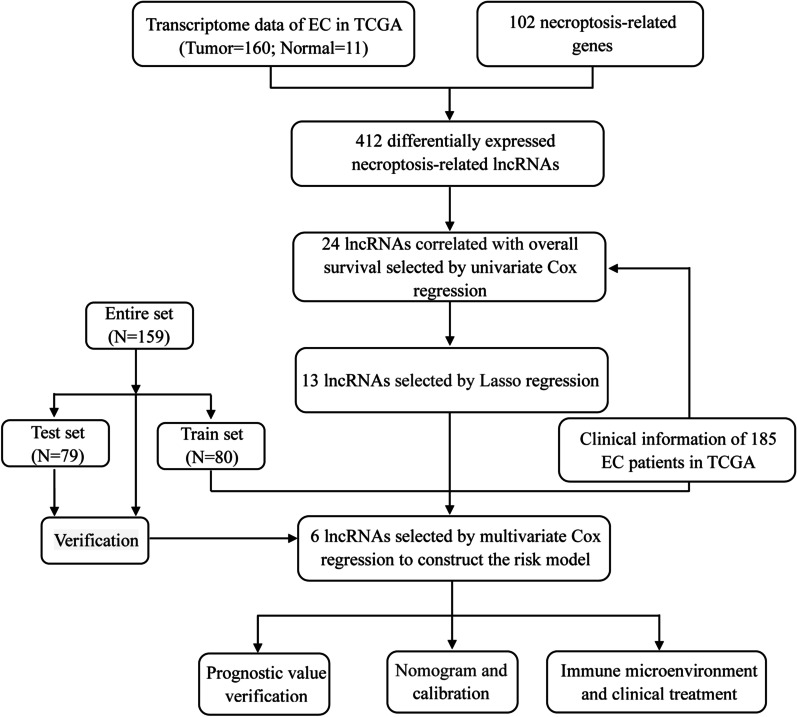
The flow diagram of the study

**Fig. 2:**
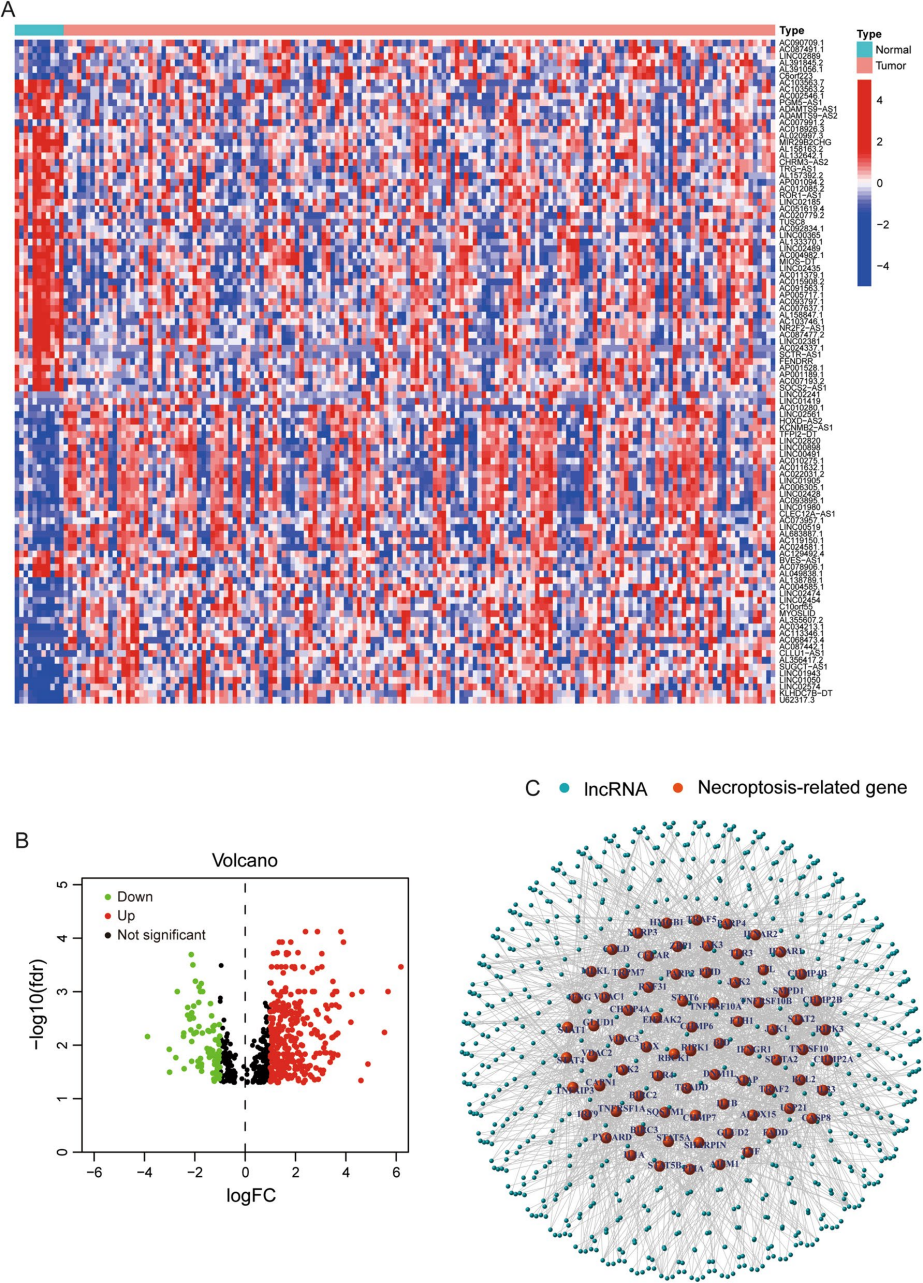
Screening necroptosis-related lncRNAs in EC. **A** 100 differentially expressed necroptosis-related lncRNAs in heat map. **B** The upregulated and downregulated necroptosis-related lncRNAs in volcano plot. **C** The network between necroptosis-related genes and lncRNAs

The flow diagram of this study was presented in Fig. 1. We collected 171 samples of transcriptome data containing 160 samples of EC tissues and 11 samples of normal esophageal tissues (Additional files 1, 2, 3, 4, 5, 6: Table S1–S6), and complete clinical data of 185 EC patients (Additional file 7: Table S7) from the TCGA database. A total of 102 necroptosis-related genes were obtained from map04217 in Kanehisa Laboratories (www.kegg.jp/kegg/kegg1.html, Additional file 8: Table S8). According to the expression of necroptosis-related genes and differentially expressed lncRNAs between EC and normal samples, we finally got 412 necroptosis-related lncRNAs (|Log_2_FC|> 1, FDR < 0.05, *p* < 0.05, Additional file 9: Table S9). Among them, there were 333 upregulated lncRNAs and 79 downregulated lncRNAs shown in volcano plot (Fig. 2B). We exhibited 100 differentially expressed necroptosis-related lncRNAs in Fig. 2A. The network between necroptosis-related genes and lncRNAs were shown in Fig. 2C and Additional file 10: Table S10.

### Construction and validation of a risk signature

**Fig. 3:**
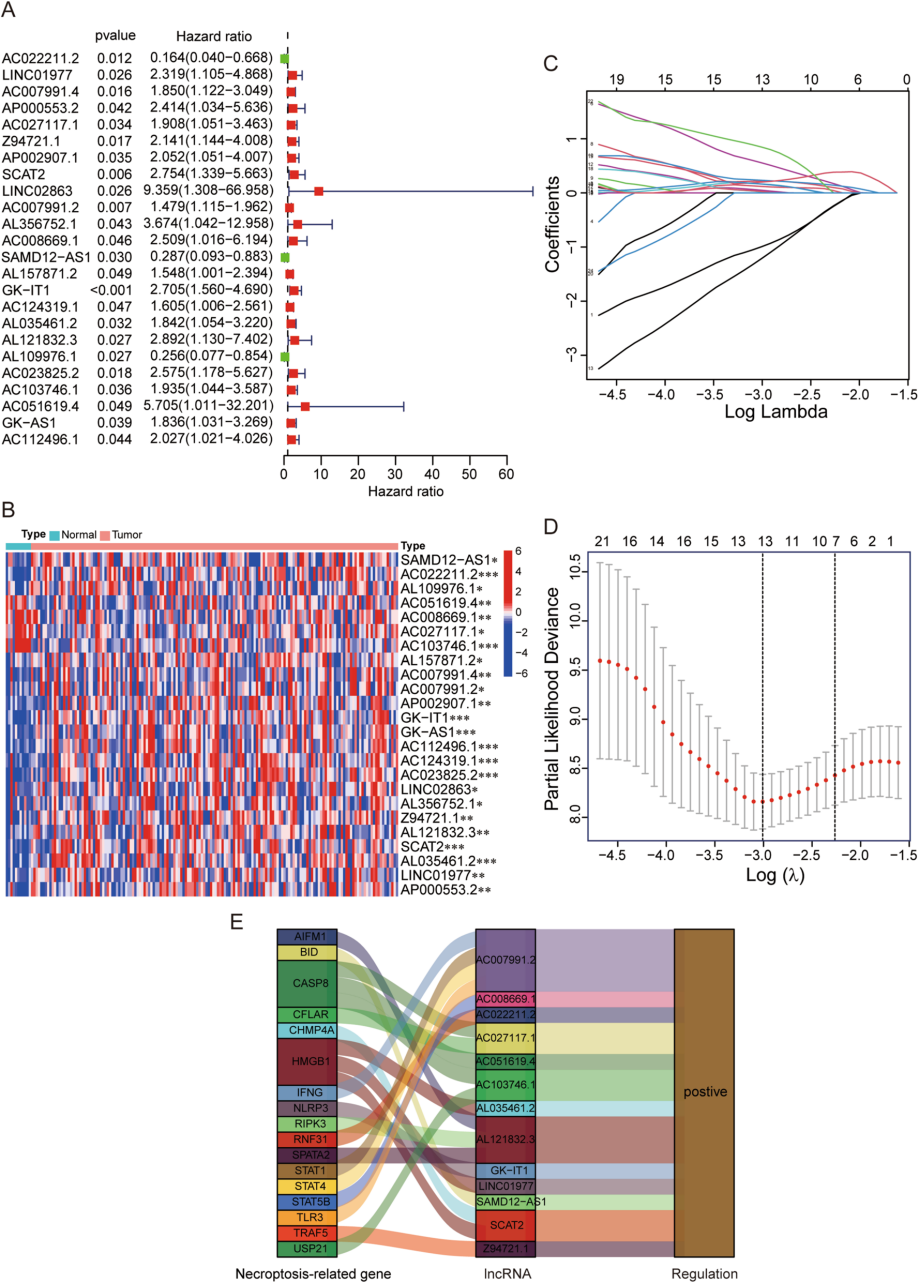
Identification of necroptosis-related lncRNAs associated with overall survival. **A** 24 necroptosis-related lncRNAs relating to overall survival of EC patients selected by univariate Cox regression analysis in forest plot. **B** The different expressions of these 24 lncRNAs in tumor and normal tissues. **C** The tenfold cross-validation for variable selection in the Lasso model. **D** The Lasso coefficient profile of 13 necroptosis-related lncRNAs. **E** The correlation between necroptosis-related genes and lncRNAs in the Sankey diagram

Based on univariate Cox regression analysis, we found 24 necroptosis-related lncRNAs significantly correlated with overall survival of EC patients (all *p* < 0*.*05, Fig. 3A), and a heat map was created to show the different expressions of these lncRNAs between tumor and normal tissues (Fig. 3B). To avoid overfitting the prognostic signature, we performed the Lasso regression on these lncRNAs and extracted 13 lncRNAs related to necroptosis in EC (Fig. 3C, D). Interestingly, we found all lncRNAs presented positive regulations with necroptosis-related genes in the Sankey diagram (Fig. 3E). Subsequently, we constructed the predictive signature composed of 6 necroptosis-related lncRNAs (AC022211.2, Z94721.1, AC007991.2, SAMD12-AS1, AL035461.2 and AC051619.4) via multivariate Cox regression analysis (Table 1). Risk score = lncRNA expression × (coef). We calculated the risk score with the formula: AC022211.2 × (− 1.7670) + Z94721.1 × (1.3917) + AC007991.2 × (0.3459) + SAMD12-AS1 × (− 2.9817) + AL035461.2 × (0.5389) + AC051619.4 × (2.1466).

**Table 1:** The 6 necroptosis-related lncRNAs signature model

lncRNA	Coef	HR	HR.95L	HR.95H	*p* Value
Z94721.1	1.391703	2.141278	1.143887	4.008323	0.017303
AC022211.2	− 1.76696	0.164307	0.040384	0.668491	0.011654
AC007991.2	0.345901	1.479099	1.115107	1.961904	0.00661
SAMD12-AS1	− 2.98166	0.287042	0.093279	0.883296	0.029529
AL035461.2	0.538899	1.842265	1.054062	3.219866	0.03197
AC051619.4	2.146606	5.705106	1.010787	32.20088	0.048596

*HR* hazard ratio, *L* low, *H* high

**Fig. 4:**
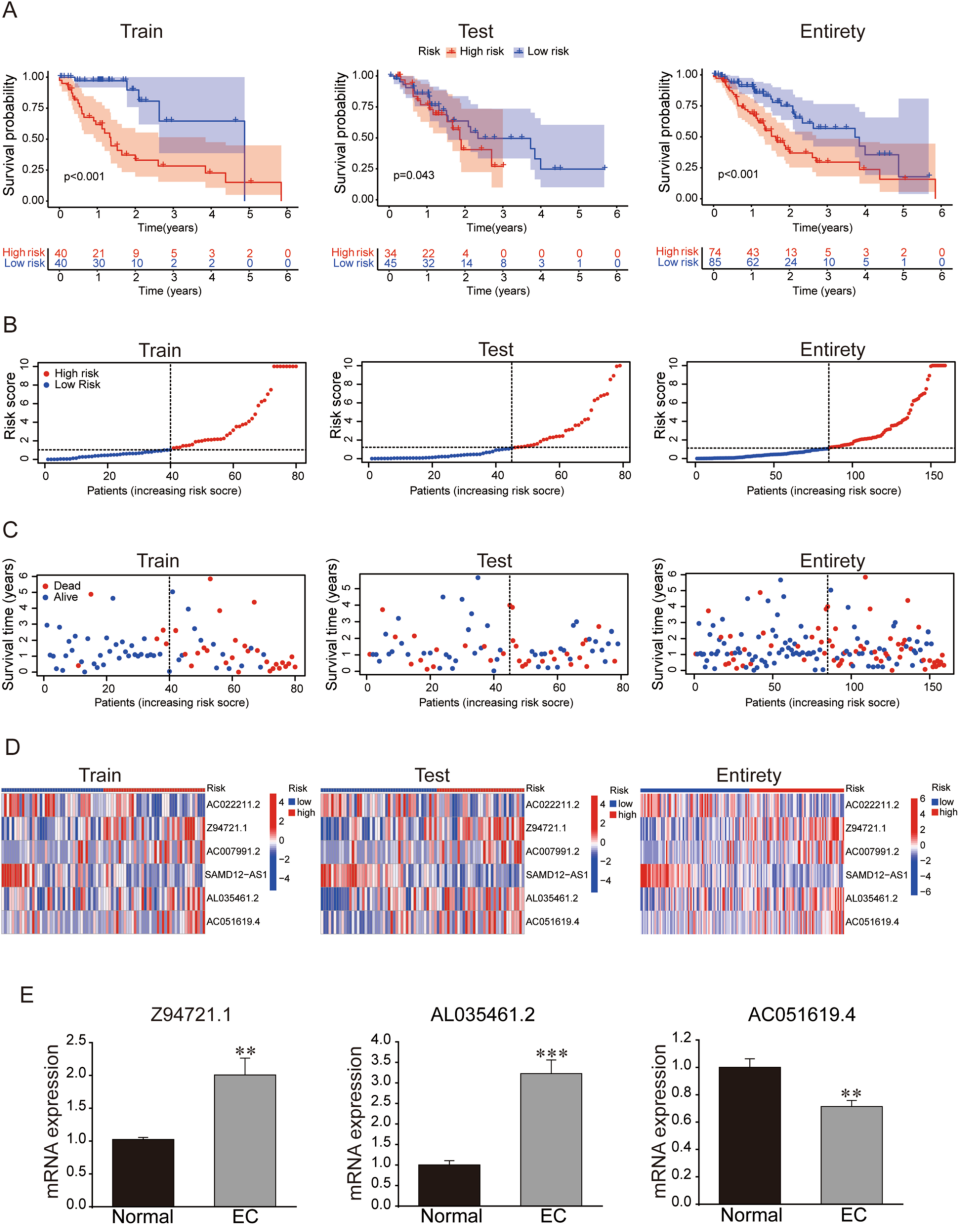
Prognosis values of the predictive signature composed of 6 necroptosis-related lncRNAs and qRT-PCR analysis. Kaplan–Meier survival curves of EC patients (**A**), the distribution of risk scores (**B**), survival time and survival status (**C**), heat maps of 6 necroptosis-related lncRNA expressions (**D**) between the high- and low-risk groups in the train (N = 80), test (N = 79), and entire (N = 159) sets. **E** qRT-PCR to detect mRNA levels of Z94721.1, AL035461.2, and AC051619.4 in normal and EC tissues. **p < 0.01, ***p < 0.001, all n = 5

According to the risk score formula, the pattern of survival time, the distribution of risk score and the survival status of these 6 necroptosis-related lncRNAs in EC patients were compared between high- and low-risk groups in the train, test and entire sets. All results indicated the high-risk group had worse prognoses (Fig. 4A–C). The relevant expressions of 6 lncRNAs showed that AC007991.2, Z94721.1, AC051619.4 and AL035461.2 were highly expressed in the high-risk group, while AC022211.2 and SAMD12-AS1 were highly expressed in the low-risk group (Fig. 4D). Furthermore, qRT-PCR analysis was performed to verify the factual accuracy of some lncRNAs in this novel risk model. The mRNA expressions of Z94721.1 and AL035461.2 were upregulated while the mRNA expression of AC051619.4 was downregulated obviously in EC tissues compared with normal tissues (Fig. 4E). These results also confirmed the reliability of the risk model.

**Fig. 5:**
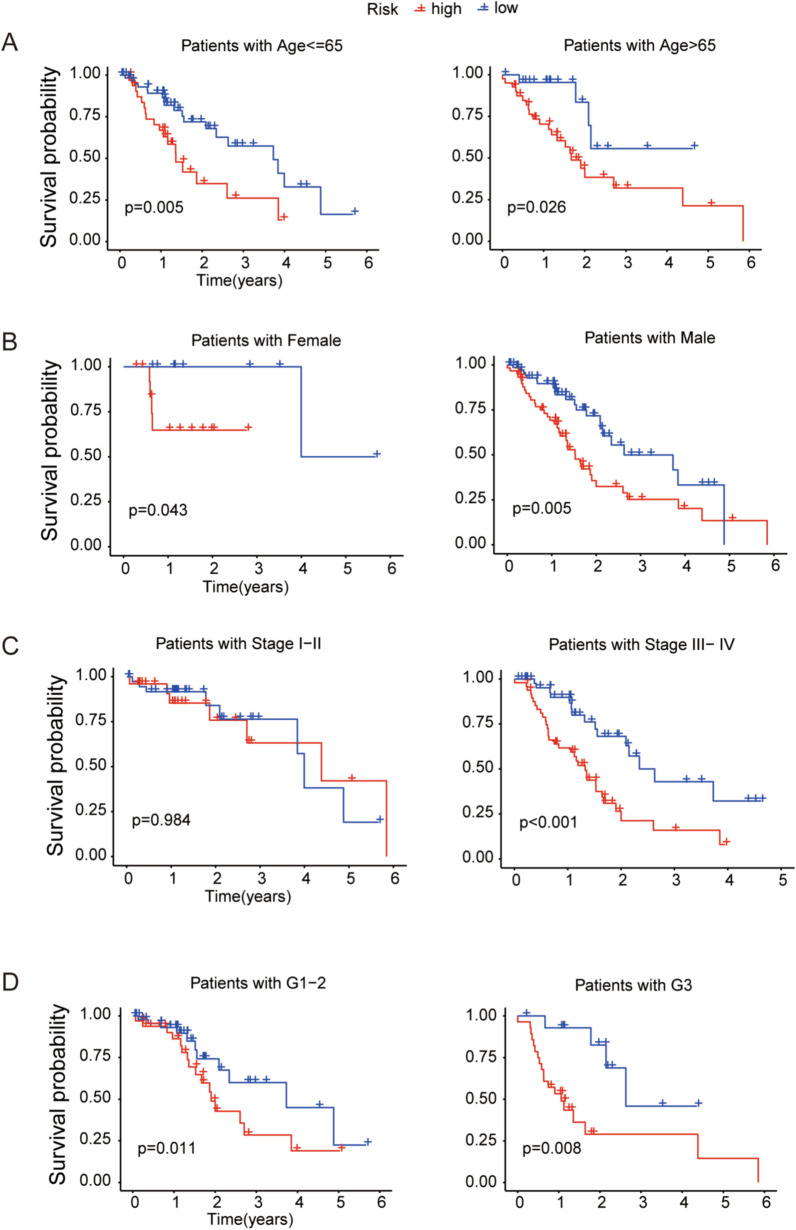
Kaplan–Meier survival curves of the risk groups sorted by different clinical features including age (**A**), gender (**B**), tumor stage (**C**) and tumor grade (**D**)

Next, we analyzed the overall survival of EC patients separated into different groups according to clinical characteristics, including age, gender, tumor grade and tumor stage. It revealed that the patients in the high-risk group had a shorter overall survival than the low-risk group except the classification of stage I-II probably due to the limited number of EC patients (Fig. 5). These results supported the predictive signature as an excellent prognostic evaluation for EC patients.

### Assessment of the risk signature

**Fig. 6:**
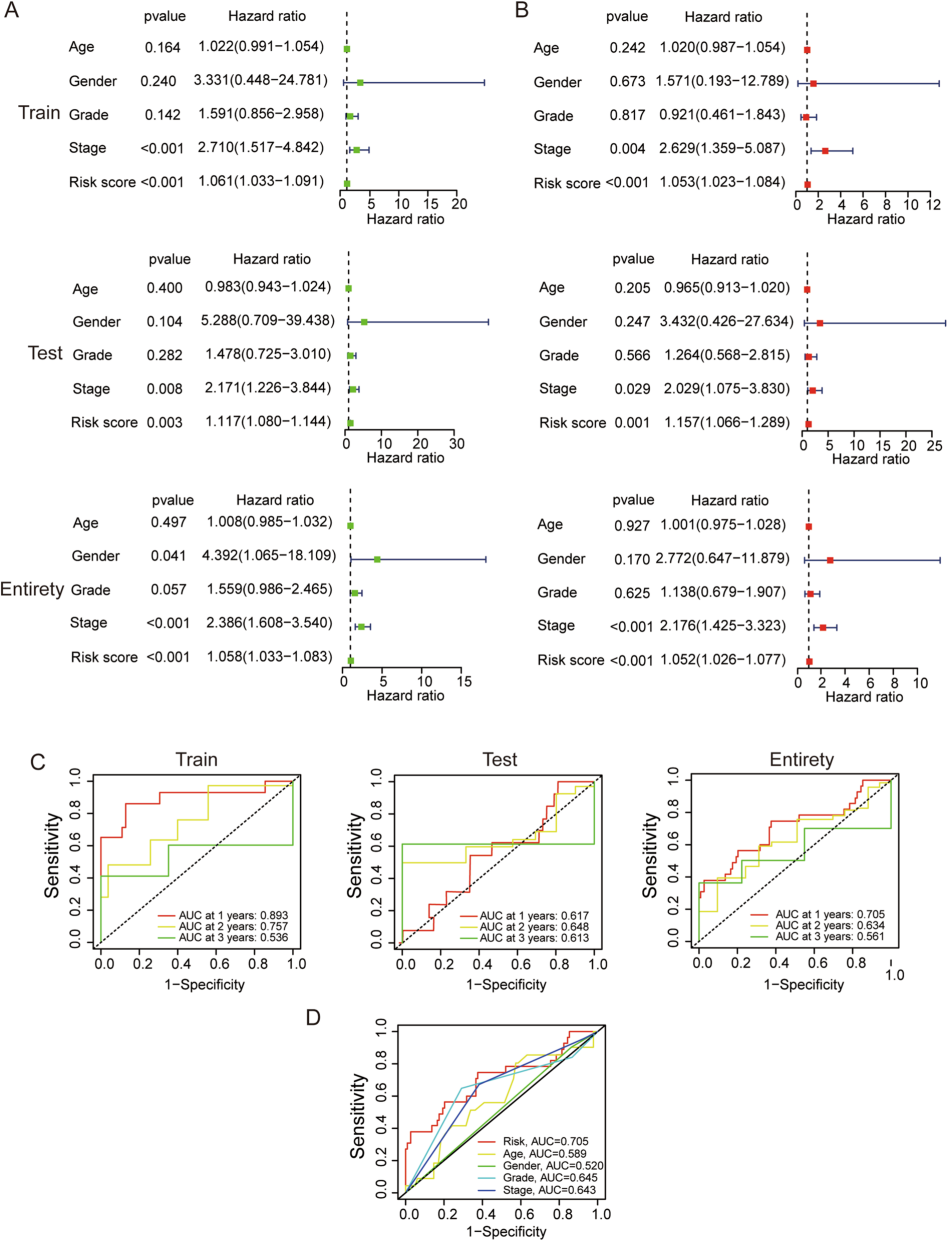
Validation of the risk signature as an independent predictor of prognosis. **A** and **B** Univariate and multivariate Cox regression analyses of clinical features and the risk score with overall survival in the train, test and entire sets. **C** Using the ROC curves to assess the prediction performance of the risk signature for the 1-, 2- and 3-year overall survival of EC patients. AUC, the area under the ROC curve. **D** Comparison of the prediction accuracy of the risk signature and clinical factors, such as age, gender, grade and stage

Whether our risk model is an independent prognostic factor for EC patients remained to be verified. The hazard ratios (HRs) of the risk score in the train, test and entire sets were 1.061, 1.117 and 1.058 respectively in univariate Cox regression analysis (all *p* < 0.05, Fig. 6A), and 1.053, 1.157 and 1.052 respectively in multivariate Cox regression analysis (all *p* < 0.05, Fig. 6B). The ROC curves were used to assess the prediction performance of the risk signature for the overall survival of EC patients, which of the outcomes were explained by the area under the ROC curve (AUC). The 1-, 2- and 3-year AUC were 0.893, 0.757 and 0.536 in the train set, 0.617, 0.648 and 0.613 in the test set, 0.705, 0.634 and 0.561 in the entire set, respectively (Fig. 6C). At the 1-year ROC of the risk score and clinical factors, the risk score in the entire set was 0.705, superior to other clinical factors (Fig. 6D). These results confirmed that the risk model could be the independent and predominant predictor for EC patients.

### Construction of nomogram and calibration

**Fig. 7:**
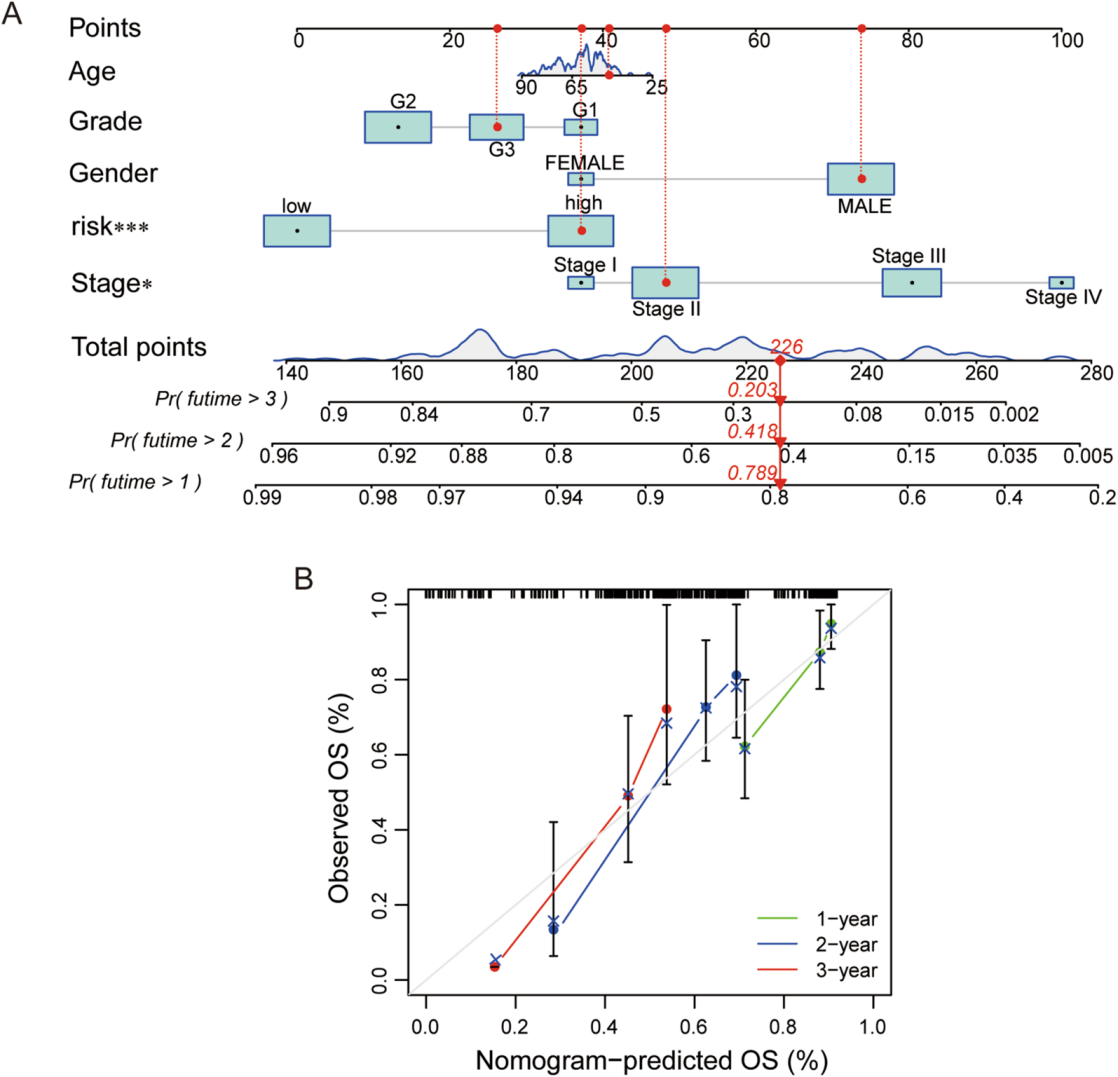
Construction of nomogram and calibration. **A** A nomogram for predicting the 1-, 2- and 3-year survival incidences of EC patients according to the risk score and clinical factors containing age, gender, grade and stage.** B** The 1-, 2- and 3-year calibration plot for proving the reliability of the nomogram. OS, overall survival

Based on the risk score and clinical features containing age, grade, gender and stage, we established a nomogram for predicting the 1-, 2- and 3-year survival incidences of EC patients (Fig. 7A). The calibration plot was used to prove that the nomogram had an excellent concordance with the prediction of 1-, 2- and 3-year survival (Fig. 7B).

### Pathway enrichment analysis

**Fig. 8:**
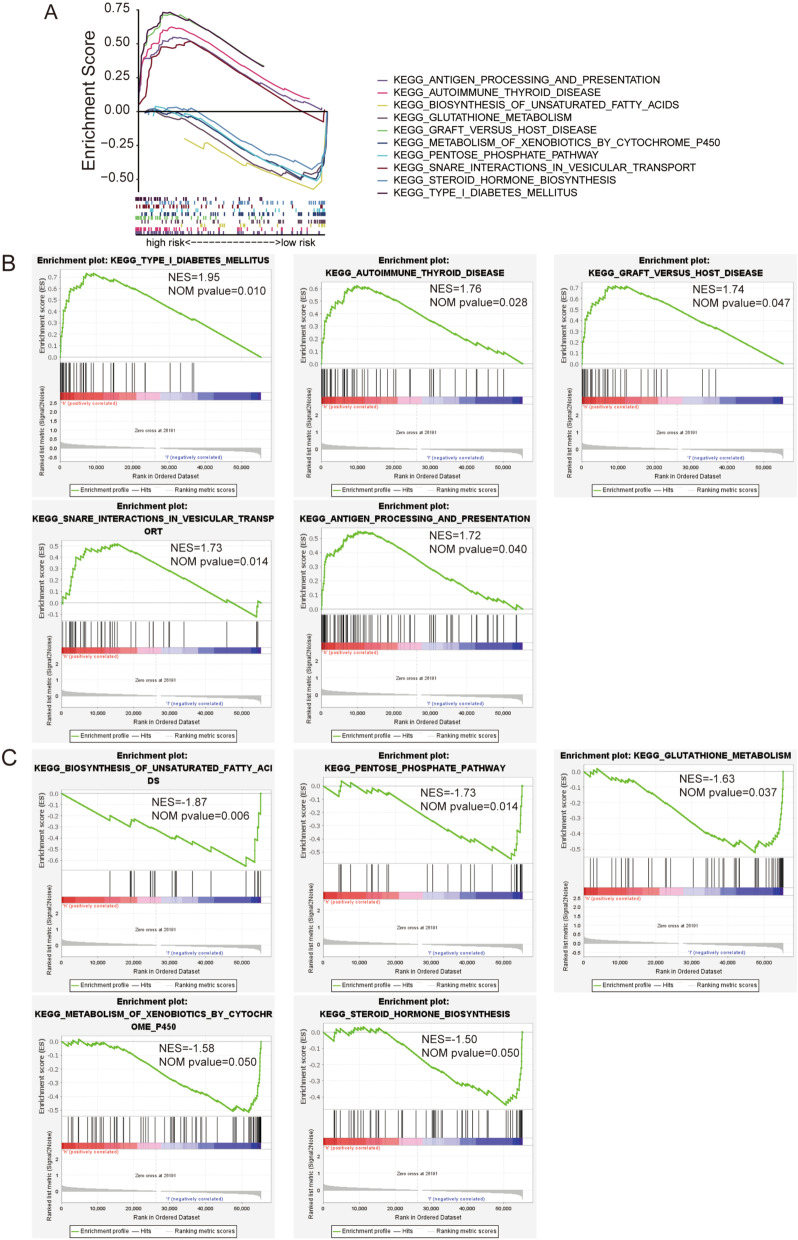
Pathway enrichment analysis via GSEA software. **A**–**C** The top 5 KEGG pathways enriched in the high- and low-risk groups. NES, normalized enrichment score. NOM, nominal

To investigate the biological functions in the risk groups, we utilized the GSEA software to explore the KEGG enrichment and pathway in the high- and low risk groups. The top 5 pathways substantially enriched in the high-risk group were type1 diabetes mellitus, autoimmune thyroid disease, graft versus host disease, snare interactions in vesicular transport, and antigen processing and presentation (all *p* < 0*.*05, |NES|> 1.5, Fig. 8A, B). And biosynthesis of unsaturated fatty acids, pentose phosphate pathway, glutathione metabolism, metabolism of xenobiotics by cytochrome p450 and ascorbate and aldarate metabolism were the top 5 pathways enriched in the low-risk group (all *p* < 0*.*05, |NES|> 1.5, Fig. 8A, C).

### Investigation of immune microenvironment and clinical treatment in the risk groups

**Fig. 9:**
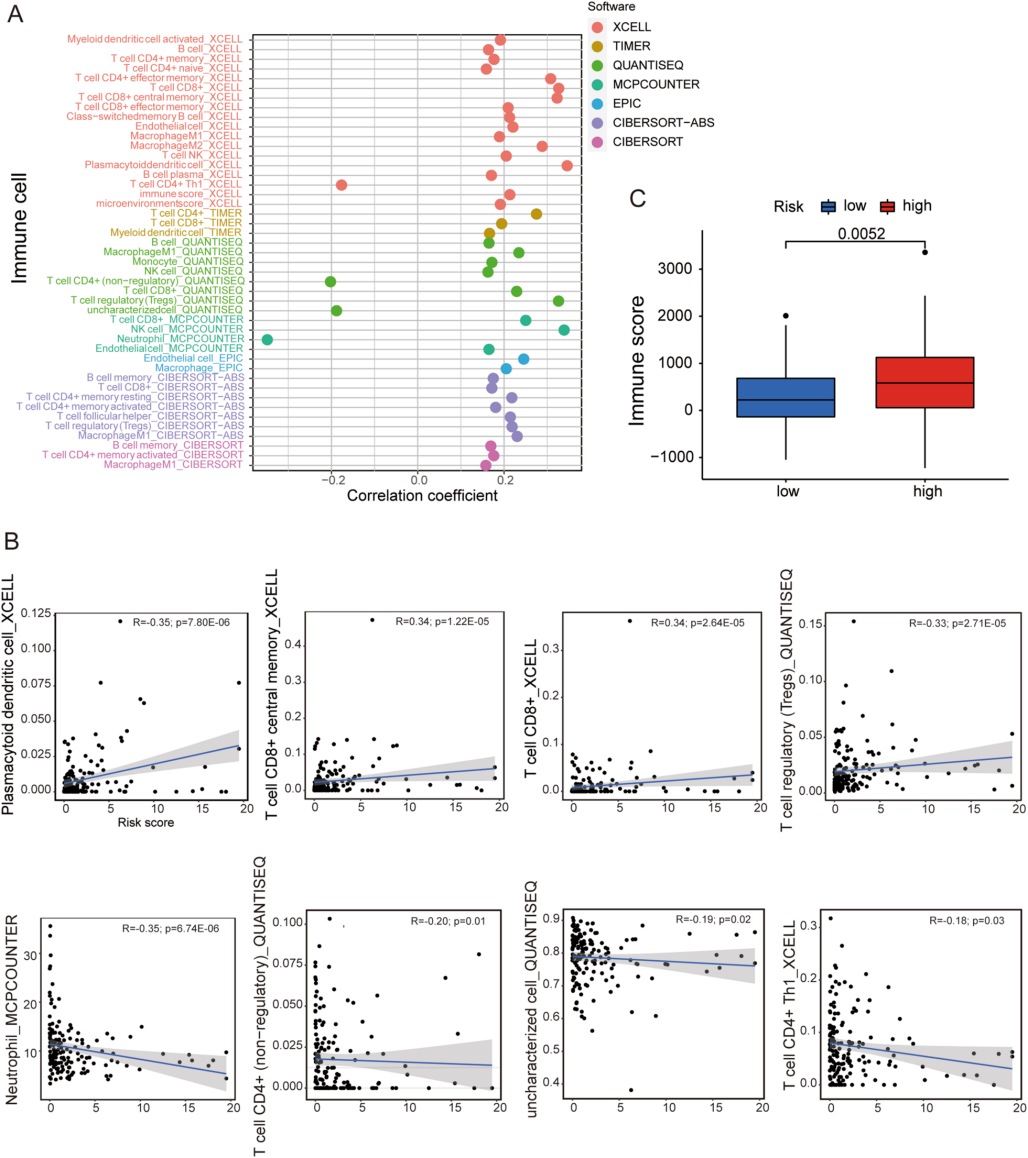
Investigation of immune microenvironment in the high- and low-risk groups. **A** and **B** The correlation between immune cells and the risk groups in a bubble chart. **C** Comparison of immune score between the high- and low-risk groups

The immune system of the body is closely related to tumors, which could eliminate tumor cells or inhibits their growth through a variety of pathways. Here we found abundant immune cells were associated with the risk model via predictive analyses of different software, including XCELL, TIMER, QUANTISEQ, MCPCOUNTER, EPIC, CIBERSORT-ABS and CIBERSORT (Fig. 9A and Additional file 11: Table S11). Most of the immune cells were positively correlated with the risk model, mainly activated in the high-risk group such as plasmacytoid dendritic cell, T cell CD8 + central memory, T cell CD8+, and T cell regulatory (Tregs), while only neutrophil, T cell CD4+ (non-regulatory), uncharacterized cell, and T cell CD4+ Th1 were negatively correlated with the risk model (all *p* < 0.05, Fig. 9A, B). Besides, we found the patients in the high-risk group had a higher immune score compared with those in the low-risk group (Fig. 9C). All of these indicated the high-risk group exhibited a higher immune infiltration status.

**Fig. 10:**
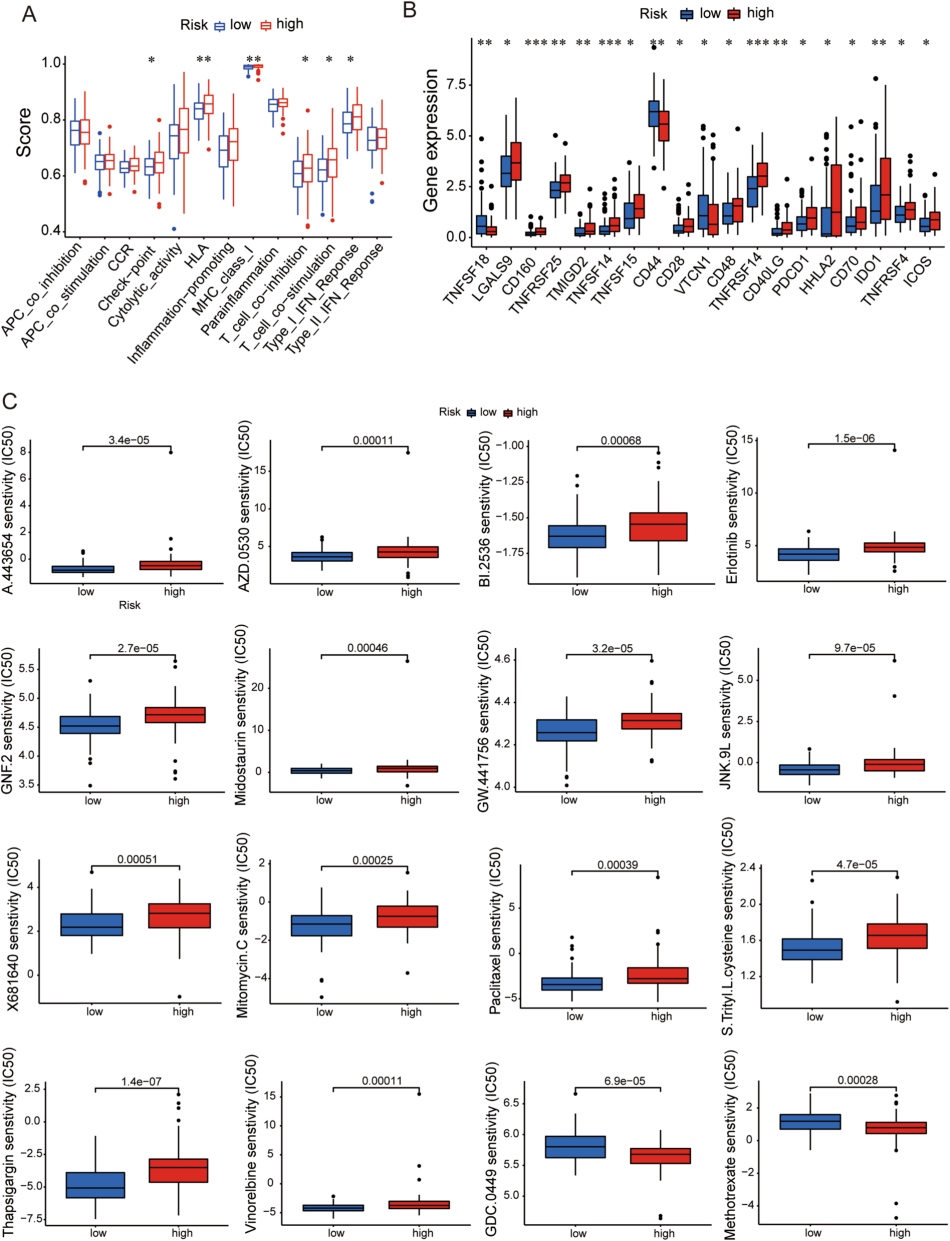
Investigation of tumor immunotherapy. **A** 6 immune functions significantly activated in the high-risk group. **B** The differences of immune checkpoint expressions between the high- and low-risk groups. **C** the prediction of potential therapeutic compounds for the risk model

Next, we investigated the immune functions associated with the risk model via ssGSEA, and the functions of check point, HLA, MHC class, T cell co-inhibition, T cell co-stimulation, and type1 IFN response exhibited higher immune scores in the high-risk group than those in the low-risk group (Fig. 10A). Most immune checkpoints also presented strong activity in the high-risk group, such as CD44, PDCD1 and IDO1 (Fig. 10B). It implied that we could choose appropriate checkpoint inhibitors to treat EC patients regrouped by the risk mode. Furthermore, the prediction of potential therapeutic drugs showed that the sensitivity to 16 chemicals in the high- and low-risk groups differed significantly. 14 compounds (Erlotinib, BI.2536, AZD.0530, A.443654, Mitomycin.C, X681640, Vinorelbine, Thapsigargin, S.Trityl.L.cysteine, Paclitaxel, Midostaurin, JNK.9L, GW.441756 and GNF.2) in the high-risk group had higher sensitivity than those in the low-risk group, and 2 compounds (GDC.0449 and Methotrexate) had higher sensitivity in the low-risk group (Fig. 10C). These studies provided a novel strategy for clinical immunotherapy of EC patients.

## Discussion

In 2020, the International Agency for Research on Cancer (IARC) released the latest global cancer data, showing that the number of new cases of EC in the world was about 604,000, the number of deaths from EC reached 544,000 [20]. Most patients with EC have no obvious early symptom, but reached advanced stage [1, 2]. Therefore, exploring molecular markers for the diagnosis, treatment and prognosis of EC becomes a top priority.

Necroptosis, one cell death pattern characterized by the activation of mixed lineage kinase domain-like protein (MLKL)/pMLKL, is mediated by receptor-interacting protein kinase 1/3 (RIPK1/RIPK3) phosphorylation signaling pathway. Recent studies suggest tumor cells that are resistant to apoptosis may be sensitive to necroptosis pathways [21, 22], hinting that the research of necroptosis and its regulation mechanism in tumor cells is expected to become a therapeutic target for EC. lncRNA as an important regulator of cell cycle, autophagy and apoptosis, plays a role in promoting or inhibiting cancers. Compared with normal cells, plenty of lncRNAs were abnormally expressed in EC cells, suggesting that lncRNA may be a biomarker for malignant tumors [6–8]. It has been reported that 16 necroptosis-related lncRNAs were constructed to predict prognosis and help make a distinction between the cold and hot tumors for improving individual therapy in gastric cancer [23]. However, the prognostic role of necroptosis-related lncRNA signature in EC has not been researched. In our study, the pattern of necroptosis-related lncRNA and the potential capability of predicting the prognosis in EC were investigated for the first time.

We got 412 necroptosis-related lncRNAs differentially expressed between the EC tissues and normal tissues from 171 transcriptome samples, in which 6 necroptosis-related lncRNAs (AC022211.2, Z94721.1, AC007991.2, SAMD12-AS1, AL035461.2 and AC051619.4) were screened out to construct the predictive signature via univariate, multivariate Cox and Lasso regression analyses. We reviewed the literature of these lncRNAs researched in tumors, and found that SAMD12-AS1 could promote gastric cancer progression via DNMT1/P53 axis [24], down-regulate P53 to promote malignant progression of glioma [25], and regulate hepatocellular carcinoma proliferation and apoptosis via the NPM1-HDM2-p53 axis [26], which indicated SAMD12-AS1 could be envisioned as a novel biomarker and therapeutic target for those cancers. AC022211.2 as one member of predictive lncRNAs signature could be the predictor for prognosis in advanced melanoma patients treated with anti-PD-1 monotherapy [27]. AC007991.2 as one member of 15 autophagy-related lncRNAs had a prognostic potential for bladder cancer, played an important role in the biology of bladder cancer [28]. For the other 3 lncRNAs, no literature has been reported to be involved in tumor studies, and the role of 6 lncRNAs in the occurrence and development of EC are still unclear, which provides more research value. In our study, qRT-PCR analysis showed upregulated Z94721.1 and AL035461.2 mRNA levels and downregulated AC051619.4 mRNA level in EC tissues compared with normal tissues, which is consistent with the expressions of them in TCGA clinical data. Combined with the survival time of EC patients, we further evaluated the reliability of the risk model via univariate, multivariate Cox regression analyses, ROC curves and the constructed nomogram, which proved the risk model as an independent prognostic factor for EC patients, but the detailed function required further exploration.

KEGG pathway analysis displayed different biological functions between the high- and low-risk groups. We found antigen processing and presentation pathway was enriched in the high-risk group. Experimental evidence supports that tumor antigen recognition by T cells is critical for antitumor immunity and that cancers can evade such immunity by immunodominance, display of immune checkpoints or immunoediting for loss of specific tumor antigens [29]. Some cancers are composed of subclonal tumor cell populations that harbor defects in antigen processing and presentation, suggesting that these cancers can’t be curable despite maximal activation of T cell immunity [30]. For those cancers without defects in antigen processing and presentation, immunogenic T cell antigens can assist in the engineering of immunotherapeutics designed to control and eradicate cancers [31, 32]. We also found pentose phosphate pathway and glutathione metabolism were enriched in the low-risk group. The pentose phosphate pathway, which branches from glycolysis at the first committed step of glucose metabolism, plays a pivotal role in helping glycolytic cancer cells to meet their anabolic demands and combat oxidative stress. Recently, several neoplastic lesions are shown to have evolved to facilitate the flux of glucose into the pentose phosphate pathway [33–35]. For glutathione metabolism, glutathione maintains cellular redox homeostasis, preserves sufficient levels of cysteine and detoxifies xenobiotics. However, glutathione metabolism plays both beneficial and pathogenic roles in a variety of malignancies. It is crucial to the removal and detoxification of carcinogens, and alterations in this pathway can have a profound effect on cell survival [36–38].

Tumor immunotherapy is a new type of therapy developed rapidly in the last decade. It mainly targets the immune system of the body, and removes tumor cells by enhancing the natural immune defense of the body against tumor and reshaping the immune microenvironment [39]. Tumor immunotherapy has brought new hope to patients with EC and other cancers. In recent years, immunotherapy has made profound breakthroughs in basic research and clinical treatment. In this study, most of the immune cells were positively correlated with the risk model, especially plasmacytoid dendritic cell. Dendritic cells are one of the most potent ‘professional’ antigen-presenting cells [40, 41]. The establishment of an immune response against cancer may depend on the capacity of dendritic cells to transfer (to capture, to process and to present) tumor antigens into regional lymph nodes where they can induce a specific response leading to tumor rejection. Dendritic cells can ingest apoptotic tumor cells and present tumor-associated antigens to T cells, leading to the generation of tumor-specific cytotoxic effect cells [42]. The immunotherapy of dendritic cells for EC is a valuable method. Dendritic cells existing in the esophageal tissues play an important role in the host's immunosurveillance against cancer as the ‘professional’ antigen-presenting cells [43].

Immune checkpoints are a series of molecules expressed on the surface of immune cells that regulate immune activation and act as a ‘brake’ in the body's immune system. When tumors occur in the body, immune checkpoints are activated and inhibit the immune function of T cells, so that tumor cells can escape immune surveillance and survive. By blocking immune checkpoints, it can activate T cells and enhance the immune response of the body, thus improving the anti-tumor ability of the body. Our findings demonstrated that most of the expressions of immune checkpoints were elevated in the high-risk EC patients compared with those in the low-risk group, such as CD44, PDCD1 and IDO1. Studies have shown CD44 overexpression is associated with a 5-year overall survival rate and could be a suitable prognostic biomarker in EC [44]. PD(L)1 inhibitors are increasingly used after first-line therapies in EC, especially among patients initially receiving chemotherapy [45, 46]. IDO1 expression is associated with an unfavorable clinical outcome in EC, supporting its role as a prognostic biomarker [47, 48]. Finally, the prediction of potential therapeutic drugs showed 14 compounds in the high-risk group and 2 compounds in the low-risk group had a higher sensitivity, which provided a good strategy for clinical treatment of EC patients.

## Conclusions

Our results supported the 6 necroptosis-related lncRNAs signature could independently predict prognosis of EC patients, and provided a theoretical basis for improving the clinical treatment of EC.

## Supplementary Information

**Additional file 1: Table S1.** 171 samples of transcriptome data containing EC and normal samples from the TCGA database.

**Additional file 2: Table S2.** 171 samples of transcriptome data containing EC and normal samples from the TCGA database..

**Additional file 3: Table S3.** 171 samples of transcriptome data containing EC and normal samples from the TCGA database..

**Additional file 4: Table S4.** 171 samples of transcriptome data containing EC and normal samples from the TCGA database..

**Additional file 5: Table S5.** 171 samples of transcriptome data containing EC and normal samples from the TCGA database..

**Additional file 6: Table S6.** 171 samples of transcriptome data containing EC and normal samples from the TCGA database..

**Additional file 7: Table S7.** The complete clinical data of 185 EC patients from the TCGA database.

**Additional file 8: Table S8.** The list of 102 necroptosis-related genes obtained from Kanehisa Laboratories (www.kegg.jp/kegg/kegg1.html).

**Additional file 9: Table S9.** The list of 412 differentially expressed necroptosis-related lncRNAs.

**Additional file 10: Table S10.** The correlation between necroptosis-related genes and lncRNAs.

**Additional file 11: Table S11.** The correlation between immune cells and the risk groups.

## Data Availability

All data analysed in this study were from public database of TCGA (https://portal.gdc.cancer.gov/) and GDSC (https://www.cancerrxgene.org/).

## Abbreviations

EC: Esophageal cancer
lncRNA: Long non-coding RNA
TCGA: The Cancer Genome Atlas
Lasso: The least absolute shrinkage and selection operator
ROC: The receiver operating characteristic
GSEA: Gene set enrichment analyses
FC: Fold change
FDR: False discovery rate
ssGSEA: Single sample gene set enrichment analysis
IC50: The half-maximal inhibitory concentration
GDSC: Genomics of Drug Sensitivity in Cancer
HR: Hazard ratio
AUC: The area under the ROC curve
IARC: The International Agency for Research on Cancer
MLKL: Mixed lineage kinase domain-like protein
RIPK1/RIPK3: Receptor-interacting protein kinase 1/3
